# Impact of Nurse-Led Asthma Intervention on Child Health Outcomes: A Scoping Review

**DOI:** 10.1177/10598405211003303

**Published:** 2021-03-24

**Authors:** Zainab Al Kindi, Catherine McCabe, Margaret McCann

**Affiliations:** 1 School of Nursing and Midwifery, 8809 Trinity College Dublin, Ireland; 2 College of Nursing, Sultan Qaboos University, Muscat, Oman

**Keywords:** nurse-led intervention program, child health outcomes, asthma programs

## Abstract

Given the leading role school nurses occupy within the school setting, they are often the most suited health care professionals to lead asthma programs. However, most school-based asthma programs have been conducted by researchers outside the school setting. Thus, we aim to determine what is currently known about the type of school nurse-led asthma intervention programs and their impact on children’s asthma-related outcomes. This article describes published literature on school nurse-led asthma intervention programs for the school-aged population using Arksey and O’Malley’s scoping review framework. A search strategy was developed and implemented in six electronic databases from 1980 to 2020. Results showed that school nurse-led asthma programs were predominantly educational interventions. Yet given the positive outcomes of school nurse-led asthma interventions reported across the articles reviewed, it is important to emphasize the leadership role school nurses assume in asthma programs, to promote more positive asthma-related outcomes in school children.

Asthma is the most common chronic condition among children, with more than a million children in the United Kingdom (Asthma UK, 2020) and more than 6 million children in the United States living with asthma (Zahran et al., 2018). Despite health care advancement, asthma prevalence and exacerbation rates continue to persist among children (Global Initiative for Asthma [GINA], 2019). Children with asthma are at risk of disability, emotional problems, and poor academic outcomes (Cicutto et al., 2013; Nurmagambetov et al., 2018). For chronic conditions such as asthma, management is achieved through medical intervention combined with self-care. As asthma is a lifelong disease, there is a demand to prioritize self-management strategies to promote positive outcomes and prevent asthma exacerbation (Isik et al., 2020).

The National Association of School Nurses (NASN) defines school nursing as a specialized public health nursing field that protects and promotes student health, enables normal development, and promotes academic success (NASN, 2016). In the school setting, school nurses are key players in asthma management. This is because they spend more time in contact with children in comparison to all other health care professionals, allowing them to develop a thorough knowledge of each child’s condition and promote self-management strategies. Additionally, their advocacy role in children’s care places them in an ideal position to identify high-risk children with asthma, plan for interventions, and evaluate programs’ effectiveness. The GINA guidelines support the implementation of effective asthma management programs for school-aged children. These programs include preventive asthma care that supports and guides school nurses’ effort to deliver asthma interventions for school communities. Consequently, school nurses focus on preventive care rather than caring for children when they are experiencing an asthma exacerbation (Halterman et al., 2018).

The impact of asthma intervention programs on children’s health outcomes using various interventions has been consistently reported in the literature (Grover et al., 2016; Horner et al., 2016; Kintner et al., 2015; Payrovee et al., 2014; Suwannakeeree et al., 2016). These interventions were delivered by research teams and required additional resources, that is, manpower and materials that could affect the sustainability of the intervention in a real-world setting. Nonetheless, several researchers found a significant improvement in school children’s quality of life (QoL; Payrovee et al., 2014), asthma management and risk reduction behavior (Kintner et al., 2015), and in self-management behavior, asthma control, and asthma knowledge (Suwannakeeree et al., 2016) following asthma intervention programs. Furthermore, other studies investigating similar interventions found fewer emergency room (ER) visits, less hospital admissions, better medication compliance, enhanced QoL, and improved asthma management self-efficacy (Grover et al., 2016; Horner et al., 2016).

Implementing school nurse-led asthma intervention programs outside of the hospital setting enables students with asthma to learn about their condition, develop asthma self-management skills, and reduce school absence and missed class time (Isik et al., 2020; Yoder, 2019). However, school-based asthma interventions are often conducted by researchers outside the school setting (Isik et al., 2019). Thus, school nurses need to be emphasized as leaders of asthma programs in a school setting and should instead conduct these interventions. To our knowledge, the literature regarding school nurse-led asthma interventions has not been systematically reviewed or synthesized. Although Isik et al. (2019) explored the effectiveness of school and community-based educational intervention programs for school-aged children and their parents, none of the studies included school nurse-led intervention programs. The current review differed because it included school-based programs that were led by the school nurse. Therefore, the objective of this scoping review was to examine and map out what is currently known about the impact that school nurse-led asthma intervention programs have on child health outcomes by including solely school-based programs that were led by school nurses. This scoping review did not include any of the studies in the review done by Isik et al. (2019).

## Method

Scoping reviews aim to identify and map the existing evidence in a specific area of research. Furthermore, they have a broader question and aim to identify research gaps in existing literature (Arksey & O’Malley, 2005). As such, a scoping review methodology was used in this article to answer the following question: What is the impact of school nurse-led asthma education programs on child health outcomes? Furthermore, the review had the following objectives:

Describe the types of school nurse-led asthma intervention programs that have been reported for the school-aged population, their impact on children’s asthma-related outcomes, and the role of school nurses in each program.
Identify the research gaps in existing literature concerning aspects of school nurse-led asthma intervention programs, particularly educational interventions.

### Inclusion/Exclusion Criteria

The population, exposure, and outcomes framework was employed to guide the selection of included studies and can be seen in Table 1 (Bettany-Saltikov, 2012).

**Table 1.** PEO Framework.

Step	Description
Population	School-aged children aged 5–18 years, their parents, school staff, nurses of any type, and other health care professionals.
Exposure	School nurse-led asthma interventions, defined as any type of asthma program (educational or noneducational) directed, provided, or administered by school nurses to enhance asthma management outcomes among students, parents, and the community and which were conducted in a school setting were included.
Outcome	Asthma management outcomes including knowledge and skills needed for asthma management; therefore, reducing health care utilization (ER visits, hospitalization) and missed school/workdays. Children’s health outcomes were broadly defined to include medication use, asthma severity, symptoms’ frequency, number of exacerbations, activity limitations, and quality of life.

*Note*. Exclusion criteria: Studies with asthma interventions who were not school nurse-led and studies outside the school setting were excluded. Case management and infection prevention studies were also excluded. PEO = population, exposure, and outcomes; ER = emergency room.

### Search Strategy

Six electronic databases were searched: Cumulative Index of Nursing and Allied Health Literature (CINAHL), PsychINFO, MEDLINE, Educational Resources Information Centre (ERIC), EMBASE, and Applied Social Sciences Index and Abstracts (ASSIA). The search was limited to articles published in the English language. The reference lists of the retrieved articles were examined but the manual search did not uncover additional eligible studies. The search was carried out between June and August 2020.

Search terms related to “asthma,” “school,” and “nurses” were adapted to maximize results from each database. These terms were combined with the Boolean operator (AND). The search strategy was designed in the CINAHL database and translated to other databases (see Table 2).

**Table 2.** Search Strategy Index Terms Used in Databases.

	Concepts/Databases	Index Term: CINAHL	Index Term: MEDLINE (EBSCO)		Index Term: PsycINFO	Index Term: EMBASE
#1	Asthma	(MH “Asthma+”)	(MH “Asthma+”)		No index term	“asthma”/exp
#2	Nurse	(MH “Nurses+”)	(MH “Nurses+”)		No index term	“nurse”/exp OR “nursing staff”/exp
#3	School	(MH “Schools+”)	(MH “Schools+”)		No index term	“school”/exp
Databases		Asthma (AND)		School (AND)		Nurse
CINAHL		(MH “Asthma+”) OR asthma*		(MH “Schools+”) OR (school* OR college* OR high school OR high-school OR preschool OR pre-school OR institute* OR junior school OR junior-school)		(MH “Nurses+”) OR (nurse* OR nursing*)
MEDLINE		(MH “Asthma+”) OR asthma*		(MH “Schools+”) OR (school* OR college* OR high school OR high-school OR preschool OR pre-school OR institute* OR junior school OR junior-school)		(MH “Nurses+”) OR nurse* OR nursing*
EMBASE		“asthma”/exp		“school”/exp OR school* OR college* OR high school OR high-school OR preschool OR pre-school OR institute* OR junior school OR junior-school		“‘nurse”/exp OR “nursing”/exp OR nurse* OR nursing*
PsychINFO		asthma*		school* OR college* OR high school OR high-school OR preschool OR pre-school OR institute* OR junior school OR junior-school		nurse* OR nursing*

### Study Selection and Data Extraction Process

**Figure 1.**
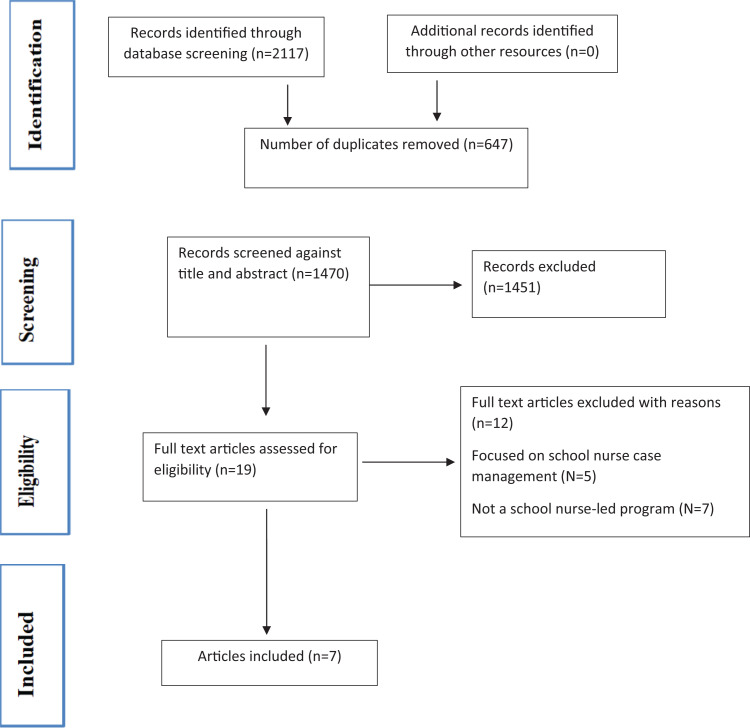
Preferred Reporting Items for Systematic Review and Meta-Analyses flowchart.

The literature review search was conducted in consultation with the university librarian. Figure 1 shows the process of study selection according to the Preferred Reporting Items for Systematic Review and Meta-Analyses guidelines. As shown, the initial database screening, which was conducted by one of the authors, resulted in 2,117 papers; 647 duplicates were removed leaving 1,470 to screen. A three-step process was followed to assess the inclusion/exclusion of articles. In the first step, screening of the title and abstracts was performed by one author using Covidence software extraction 2.0 (Covidence GmbH, Australia). Following a review of titles and abstracts, 19 papers went forward for full-text review. On the second step, the other two authors independently reviewed the full text of the 19 articles to determine alignment with the inclusion criteria. Of these, 12 studies were excluded because they focused on school nurse case management (*N* = 5) and were not a school nurse-led intervention (*N* = 7). Overall, seven studies were included in the review. There were no disagreements between the evaluators when selecting and extracting the data. The third step was to review the bibliography of the identified articles; no additional articles were identified.

Data from the included studies were extracted into an evidence table that included author, year, country, aim, design, population/sample, nature of the intervention, outcome assessed, results, and measurement tools (see Table 3).

**Table 3.** Characteristics of the Included Studies.

#	Citation, Country	Objective	Design	Population Sample	Nature of Intervention	Tools	Outcomes Results
1	Persaud et al., 1996, United States	To examine the effectiveness of an asthma education program on children and their parents.	RCT Level I ^a^	36 Children with asthma (8–12 years old, mean age = 10.2) randomly assigned to an intervention group (18) and (18) a control group.	SN attended two 4-hr in-service sessions to improve asthma management knowledge and skills. After the training, SN delivered a 20-min individual training session to children over 8 weeks. Children were divided into an intervention group and a control group. The intervention group received teaching sessions and the child’s weekly progress was recorded on a checklist. The control group received regular care but no asthma education.	Five standard questionnaires: Children’s asthma knowledge: 20-items; Parent’s asthma knowledge: 55-items; Child’s asthma attitude: 28-items; Child health locus of control: 20-items; Functional status: 28-items.	Children’s asthma knowledge (*p =* .9); health locus of control (*p =* .17); attitudes toward asthma (*p =* .3); functional status (*p* = .8); school absenteeism (6.4 days vs.7.6 days); and ER visits (*p* < .05); Parent’s asthma knowledge (*p* = .54).
2	Salisbury et al., 2002, United Kingdom	To compare a nurse-led clinic in schools versus care in general practice for adolescents with asthma.	An RCT in four schools, a parallel observation study in 2 schools Level I	*N* = 490 adolescents with asthma, mean age = 13Age range: NA Intervention (156), control (149)	Adolescents with asthma were individually randomized to receive a review of their asthma status at school (school clinic group *n*= 156) or in general practice (practice care group *n*= 149). A parallel observational comparison between adolescents in the practice care group of the randomized trial and pupils in two control schools (control school group) was done.	Pediatric quality of life questionnaire; Asthma knowledge and attitude quiz.	Primary: Attendance for an asthma review (*p* < .001); Symptom control (*p* = .42); Quality of life (*p* = .63). Secondary: Knowledge (*p* = .001) and attitude (*p* = .007); Inhaler technique (*p* < .001); use of steroids (*p* = .60); school absence (*p* = .78); PFM (*p* = .36).
3	D. M. Carpenter et al., 2016, United States	To test whether a tailored inhaler technique video intervention (1) could be feasibly implemented by school nurses and (2) improve the inhaler technique of children with asthma. Duration: 1 month Theory: No	Quasi-experimental design Level III	*N* = 25 children with asthma (age 7–17, mean age =11.5)	SN were trained on MDI technique using a validated training process. Then, SN recruited asthmatic children measuring children’s inhaler technique before and after watching tailored inhaler technique videos. Immediate feedback was given to the child and a record of child performance was shown. One month later, reassessment was done	Validated checklist	Inhaler technique Significant improvement with spacer (*p* = .03), without spacer (*p* = .01)
4	Harrington et al., 2018, United States	To investigate whether high adherence to ICS asthma preventive treatment among students with persistent asthma was achievable by school nurse supervised asthma therapy. Duration: 60 days Theory: No	Pilot, prospective, RCT Level I	*N* = 45 children with asthma in elementary and middle school (5–12 years old, mean age = 8). Intervention group = 21; Control group = 25.	Directly observed therapy by SN every day the school was in session and every evening by parents on school days. Every morning and evening on weekends and holidays.		The total proportion of ICS morning doses (*p* = .11). School absence (*p* = .17). Health care utilization: ER visits (*p* = .71), hospitalization (*p* = .29) QoL: Family adjustment (p = .03), Less functional limitation (*p* = .04).
5	Trivedi et al., 2018, United States	To examine the impact of a novel, school nurse-supervised asthma therapy program on health care utilization. Duration: 24 months Theory: No	Quasi-experimental, time series design	*N* = 62 children with asthma from 44 schools (age range: NA, mean age is 10.5)	SN direct observation of daily preventive asthma medication	School records	ER visits (*p* < .001), hospital admissions (*p* < .001), school absences (*p* = .28), and rescue medication use (*p* < .001).
6	Simoneau et al., 2020, United States	To evaluate the effectiveness of a five-element school nurse-led asthma management program in providing asthma care and reducing school absenteeism.	Quasi-experimental Level III	15 School nurses *N* = 251 students with asthma from elementary schools (age range (6–12), mean age 8.7.	28 School nurses trained through an on-site, one-on-one 30-min session, provided with training manual and instruction on five elements (asthma risk assessment, control, education, medication, and communication with the medical provider). Once trained, SN (*n* = 15) implemented the program in their schools based on a needs assessment of each student (two academic years). Ten SN surveyed about confidence in asthma management.	Retrospective review of school records.	School absence (*p* < .001), Inhaler technique (*p* < .001) School nurse satisfaction (92% were satisfied).
7	Isik et al., 2020, United States	To examine the effectiveness of a theoretically based school nurse-led asthma intervention on asthma symptoms, self-management, peak flow meter usage, daily activities, and school absences in children 7–12 years old. Theory: Orem’s self-care theory	An RCT, repeated measures design Level I	*N* = 37 asthmatic children (7–12 years old, mean age = 9) from eight primary schools.	The treatment group received the asthma intervention composed of theoretically based school nurse-led asthma intervention, which comprised six weekly 30-min group lessons, PFM for personal use, and hands-on experiences. The control group continued to receive usual care. Data collection points: At baseline, 6 weeks, and 12 weeks.	Asthma Control Questionnaire (ACQ) Pediatric Asthma Quality of Life Questionnaire (PAQLQ)	Symptoms (*p* < .001), asthma control with a peak flow meter usage (*p* < .001), daily activities (*p* < .001). School absence (*p* = .17).

*Note*. SN = school nurse; NA = not applicable; ER = emergency room. ^a^ Refer to level of evidence in Table 4.

## Results

The purpose of this review was to identify the current evidence on the impact of school nurse-led asthma intervention programs. The school nurse’s role in the included studies varied between providing educational interventions for asthmatic children (D. M. Carpenter et al., 2016; Isik et al., 2020; Persaud et al., 1996; Simoneau et al., 2020), supervising the administration of preventive asthma medication (Harrington et al., 2018; Trivedi et al., 2018), and running a school asthma clinic (Salisbury et al., 2002).

### Study Characteristics

All included studies (*N* = 7) discussed an asthma program led by a school nurse (D. M. Carpenter et al., 2016; Harrington et al., 2018; Isik et al., 2020; Persaud et al., 1996; Salisbury et al., 2002; Simoneau et al., 2020; Trivedi et al., 2018). Furthermore, all reported at least one of the asthma health-related outcomes. The seven studies combined represented a total of 946 school-aged children and 15 school nurses. The sample size ranged from 25 to 490. The target population of the included studies was children alone (D. M. Carpenter et al., 2016; Harrington et al., 2018; Isik et al., 2020; Salisbury et al., 2002; Trivedi et al., 2018), children with their parents (Persaud et al., 1996), or children and school nurses (Simoneau et al., 2020). Notably, none of the studies included in this review indicated the qualification of the school nurses who led the interventions.

The intervention programs led by school nurses were conducted in the school setting. Six studies were conducted in the United States and one in the United Kingdom (Salisbury et al., 2002). The research study designs used in the included studies were randomized controlled trials (RCTs; *N* = 4; Harrington et al., 2018; Isik et al., 2020; Persaud et al., 1996; Salisbury et al., 2002) or quasi-experimental designs (*N* = 3; D. M. Carpenter et al., 2016; Simoneau et al., 2020; Trivedi et al., 2018). Table 4 shows the evidence level (Melnyk & Fineout-Overholt, 2015). The discussion of studies in this review is grouped into the following categories: (1) school nurse asthma education intervention, (2) school nurse supervised asthma therapy, (3) school nurse asthma clinic, and (4) asthma health-related outcomes.

**Table 4.** Melnyk Levels of Evidence.

Level 1—Systematic review and meta-analysis of randomized controlled trials, clinical guidelines based on systematic reviews or meta-analyses
Level 2—One or more randomized controlled trials
Level 3—Controlled trial (no randomization)
Level 4—Case-control or cohort study
Level 5—Systematic review of descriptive and qualitative studies
Level 6—Single descriptive or qualitative study
Level 7—Expert opinion

### School Nurse Asthma Education Intervention

Four studies evaluated school nurse-led asthma education programs with two being RCTs (Isik et al., 2020; Persaud et al., 1996) and two quasi-experimental studies (D. M. Carpenter et al., 2016; Simoneau et al., 2020). The format of the education programs varied to include structured educational sessions (Isik et al., 2020; Persaud et al., 1996; Simoneau et al., 2020) and a tailored video program (D. M. Carpenter et al., 2016). The asthma education session led by school nurses was delivered to a group of participants in a face-to-face setting (group format) in two studies (D. M. Carpenter et al., 2016; Isik et al., 2020; Simoneau et al., 2020) and a one-on-one session (individual format) in two studies (Persaud et al., 1996; Simoneau et al., 2020).

The participating school nurses in Persaud et al.’s (1996) study attended a 4-hr training session to improve their knowledge and skills before they delivered the education session to children. They received a theoretical session covering asthma pathophysiology, management strategy, and medication use. School nurses also received a practical demonstration on peak flow monitoring, inhaled medication, and the use of spacer devices. They were also introduced to the following educational strategies: how to communicate with the child, conduct interviews and role-plays, and provide positive reinforcement. Following the 4-hr training session, school nurses provided a weekly one-on-one, 20-min session for each child for 8 weeks. Children were taught asthma self-management principles and peak flow monitoring. Before the education sessions and following the consent process, children were randomly assigned to the intervention group (*N* = 18) and the control group (*N* = 18). Both groups had a preintervention appointment where they received a physical examination and pulmonary function test by a pediatrician. However, the intervention group received education sessions by the school nurse, while the control group received no education. There were two data collection points, at baseline and immediately following the 8-week session.

Similarly, D. M. Carpenter et al. (2016) provided a validated training program to school nurses before these school nurses delivered the education sessions to school children. However, D. M. Carpenter et al.’s (2016) study focused on the inhaler technique. School nurses watched a demonstration video on inhaler use by three children. Then, they viewed 18 incorrect inhaler techniques and used a certified respiratory therapist checklist to score each technique; thereafter, they received feedback on each video. After the training, school nurses recruited a convenience sample of seven asthmatic children (four elementary, two middle school, and one high school). Children demonstrated their inhaler technique and school nurses evaluated the demonstration using a checklist score. The children’s demonstrations were video-recorded and entered into a tailored video software program. Children then viewed a correct inhaler technique by one of the video characters of their choice (there were six characters with different genders and ethnicities). The children then watched their 1–2-min tailored video with systematic feedback on each step demonstrated. In addition, children were praised for each correct step with positive statements. One month later, school nurses asked children to demonstrate the inhaler technique and evaluated them by using the same validated checklist to score their technique. This was the only follow-up point after the intervention.

Simoneau and colleagues (2020) investigated the effectiveness of a five-element school nurse-led asthma management program in providing asthma care and reducing school absenteeism. In this study, the school nurse-led asthma intervention extended beyond a structured education program. Researchers trained 28 school nurses on five program elements (asthma risk assessment, control, education, medication, and communication) with the medical care provider. Once trained, school nurses conducted the five-element program in their school setting. They continued to enroll students throughout the two academic years (2015–2016 to 2016–2017). An area of concern in this study is that school nurses could choose elements to deliver based on a needs assessment of each student with asthma. This led to the limited implementation of specific elements such as communication with primary care providers where school nurses communicate concerns or ask questions to the child’s primary care provider. The most utilized element was the inhaler technique task. There were 102 students with asthma who completed two inhaler technique assessments over the 2 academic years: one at baseline and one at the end of the academic year.

Isik et al. (2020) investigated the effectiveness of a theoretically based school nurse-led asthma intervention on symptoms, asthma self-management with peak flow meter (PFM) usage, daily activities, and school absenteeism among elementary school children aged 7–12 years. Participants were randomly assigned to receive a school nurse-led asthma intervention (treatment *N* = 37) or usual care (control *N* = 36). The intervention group received a school nurse-led asthma group session over 6 weeks. Topics covered were (1) pathophysiology, (2) PFM, (3) symptom identification and asthma action plans, (4) medication and delivery devices, (5) triggers identification and prevention as well as breathing exercises, and (6) individualized discussion and self-management strategies. To improve class interaction, researchers utilized hands-on practice, problem-based learning, case studies, storytelling, role-plays, and class discussions. The treatment group received a PFM with a personal peak flowchart and a spacer. The control group received usual care, which refers to receiving their medication according to the action plan prescribed by the child’s doctor. Data collection took place three times: at baseline, 6 weeks, and 12 weeks.

### School Nurse-Supervised Asthma Therapy

Two studies examined school nurse-supervised asthma therapy (Harrington et al., 2018; Trivedi et al., 2018). In an RCT, Harrington et al. (2018) assessed school nurses’ administration of asthma therapy to students with persistent asthma (*N* = 21) and its impact on asthma-related health outcomes. The school nurse administered a daily dose of the inhaled corticosteroids (ICS) every morning at school to those assigned to the intervention group (*n* = 18). The clinician prescribed administration of ICS at home every evening of a school day, and every morning and evening during holidays. Parents did home medication administration. For the control group (*n* = 25), the ICS doses were prescribed to be administered daily by parents at home every morning and evening for the 2-month study period. There were two data collection points: at baseline and 60 days after enrolment. Structured telephone follow-up interviews were conducted with parents to assess ICS doses and parental reported asthma symptoms and disability during the study period.

Similarly, Trivedi et al. (2018) investigated the outcome of health care utilization after a school nurse-supervised asthma intervention that included daily directly observed ICS medication administration. The difference in this study is that the school nurse administered both the morning (at 8:00 a.m.) and evening doses (at 2:00 p.m.) at school. There were 84 enrolled children with asthma. As part of the program, school nurses provided training to participants on the correct inhaler and spacer techniques, but there was no reporting on how often instructions were given or how often these skills were followed up on. There was a retrospective assessment of asthma-related ER visits, asthma-related hospital admissions, and school absenteeism 1 year before enrolment and 1 year after enrolment.

### School Nurse Asthma Clinic

Salisbury et al. (2002) conducted an RCT of a nurse-run asthma clinic in four secondary schools. Asthmatic adolescents at these schools were randomly assigned to receive a review of their asthma status at school (school clinic group) or in general practice (GP; practice care group). The nurse-run asthma clinic took place weekly in each of the four schools. The school clinic group received the same care as the practice care group; however, the discussion was tailored to the needs and interests of adolescents. Follow-up was done at the school asthma clinic 1 month and 6 months after the initial assessment. Students in the control group were invited by their practice to attend an asthma review that could be provided by a practice nurse or a doctor, according to the usual practice.

### Asthma-Related Health Outcomes

The focus of this scoping review is to discuss the available evidence in relation to the impact of school nurse-led asthma programs on children’s health-related outcomes. The measurement of different outcomes varied and was complex due to the incomplete data reported by some papers. The outcomes reported included: medication use, inhaler technique, and PFM use, symptoms, school absence, QoL, health care utilization, and asthma knowledge.

#### Medication use

Three studies evaluated medication usage among asthmatics in a school setting after a school nurse-led asthma program (Harrington et al., 2018; Salisbury et al., 2002; Trivedi et al., 2018). Trivedi et al. (2018) found a significant decline in the number of asthma rescue medication (beta agonist) refills between the pre-and postintervention periods (*p* < .001). Harrington et al. (2018) reported similar significant findings as the intervention group received 91.7% of the expected morning dose of ICS at school by the school nurse (that is more than the hypothesized percentage of 80%). Contrastingly, Salisbury et al. (2002) reported no significant differences in the number of adolescents prescribed ICS (*p* = .89) or daily ICS use (*p* = .60) following the implementation of a school nurse-led asthma program.

#### Inhaler technique and PFM use

Two studies assessed the inhaler technique among students with asthma (D. M. Carpenter et al., 2016; Salisbury et al., 2002). D. M. Carpenter et al. (2016) evaluated a school nurse-led tailored video intervention to improve inhaler techniques among asthmatic children. The number of correct steps increased significantly from baseline after watching the video (*p* = .03). Carpenter and colleagues reported that the steps children most commonly missed were exhaling normally before taking a deep breath, shaking their inhaler four to six times, and holding their breath for 10 s after inhaling the medicine. In this study, children significantly sustained inhaler technique at the 1-month follow-up. Likewise, adolescents with asthma in the intervention group demonstrated higher inhaler scores (*p* < .001) than their peers randomized to the control group in the study by Salisbury et al. (2002).

There was a lack of PFM assessment in the included studies with only two studies highlighting this outcome (Persaud et al., 1996; Salisbury et al., 2002). Persaud et al. (1996) did not assess changes in PFM reading; however, they reported that knowing the baseline PFM reading facilitated decision making by school nurses on when to seek medical help or when to advise the child to use an inhaler. Salisbury et al. (2002) assessed PFM readings before and after the intervention, reporting no differences in PFM reading (*p* = .36) between the groups.

#### Symptoms

Two studies assessed the impact of school nurse-led asthma programs on symptom frequency (Isik et al., 2020; Salisbury et al., 2002). While Salisbury et al. (2002) reported no differences in the level of symptoms (*p* = .42) for the intervention group (adolescents) attending a school nurse asthma clinic, Isik et al. (2020) reported a statically significant difference in asthma symptoms among the treatment group after a theoretically based school nurse-led asthma intervention (*p* < .001).

#### School absence

The outcome of school absenteeism was measured using school records or self-reported data in six of the included studies (Harrington et al., 2018; Isik et al., 2020; Persaud et al., 1996; Salisbury et al., 2002; Simoneau et al., 2020; Trivedi et al., 2018). Overall, these studies reported a reduction in the number of missed school days among school-aged children after the school nurse-led intervention. Persaud et al. (1996) reported that 5 months following an asthma education program delivered by school nurses, there was a decline in the number of missed school days, but there was no significant difference between the intervention and the control group (6.4 and 7.6 days, respectively). The same result was supported by Salisbury et al. (2002) and Trivedi et al. (2018) who also reported a nonsignificant decline in asthma-related school absence between students in the treatment and control group.

Simoneau et al. (2020) reported that students with asthma who engaged in a school nurse-led asthma program (easy breathing for schools) had a 25% reduction in their absenteeism rate compared to students without asthma who did not engage. This translated into three to four gained school days annually. Similarly, Isik et al. (2020) reported that school absence decline was not statically significantly different between groups (*p* = .179), yet the treatment group missed fewer school days.

#### QoL

In our review, three studies examined QoL as an outcome (Harrington et al., 2018; Persaud et al., 1996; Salisbury et al., 2002). Salisbury et al. (2002) assessed the differences in QoL among asthmatic adolescents assigned to the school clinic or GP care. There was no significant difference in the QoL score among the groups (*p* = .63).

The study by Persaud et al. (1996) assessed child health locus of control using an instrument that measured children’s perception of the extent to which their health was primarily affected by their action versus outside forces. This study also assessed children’s feelings about themselves using an asthma attitude survey. While differences existed between the intervention and control group, these were not significant and would require a larger sample to approach significance.

Harrington et al. (2018) assessed multiple measures of QoL in terms of functional limitation, family adjustment, and medical interference. There were significantly less functional limitations (*p* = .04), required adjustment to family life (*p* = .03), and sleep loss due to asthma (*p* = .04) after the school nurse-supervised asthma therapy. Overall, there was a statistically significant difference in QoL in the treatment group compared to the control group (*p* < .001).

#### Health care utilization

The impact of school nurse-led asthma programs on health care use includes ER visits, unscheduled visits to physicians, and/or hospitalization. Out of the seven included studies, three studies explored the impact of a nurse-led asthma program on the number of ER visits (Harrington et al., 2018; Persaud et al., 1996; Trivedi et al., 2018). Persaud et al. (1996) reported that the percentage of students who attended the ER due to an exacerbation of their asthma was significantly higher in the control group (50% *N* = 9) than the intervention group (22%, *p* ≤ .05). When the age of onset was controlled, however, the association between the number of ER visits per child and the educational intervention was not statistically significant.

Trivedi et al. (2018) also found a significant reduction in asthma-related health care utilization in a recent study. The authors reported a decrease in asthma-related ER visits and asthma-related hospitalization over a 1-year follow-up period after enrolment in a school nurse supervised asthma therapy. The ER visits declined 37.5% from the preintervention mean of 0.8 to a postintervention mean of 0.3 visits (*p* < .001). Asthma-related hospital admission showed a significant decline as well from a preintervention mean of 0.3 admissions to a postintervention mean of 0 admissions (*p* < .001). Contrastingly, Harrington et al. (2018) reported no difference in unscheduled ER visits or hospitalization as the school nurse-supervised asthma therapy was administered over 60 days.

#### Asthma knowledge among children and their parents

Only two studies in this review assessed the knowledge of children after an asthma program was delivered by a school nurse (Persaud et al., 1996; Salisbury et al., 2002). Persaud et al. (1996) found that there was no statistically significant difference in the knowledge score among children (*p* = .9) and their parents (*p* = .54) after program implementation. This was the only study that addressed parent’s knowledge alongside children’s knowledge. In contrast, Salisbury et al. (2002) found that asthmatic students attending school clinics (treatment group) had a greater knowledge of asthma compared to students in the practice care group (control group; *p* = .001).

## Discussion

As has been illustrated, nurses play a leading role in the school setting, placing them in an optimal position to lead programs in asthma management. However, most school-based asthma programs were conducted by researchers outside the school setting; thus, highlighting a gap in the literature. We, therefore, aimed to determine what is currently known about the type of school nurse-led asthma intervention programs and their impact on children’s asthma-related outcomes by conducting a scoping review.

### Types of School Nurse-Led Intervention Programs and Their Outcomes

Our scoping review found that school nurse-led asthma programs had a positive impact on children’s asthma health-related outcomes, which include medication use, reduction in school absenteeism, reduction in health care utilization, improved asthma knowledge, and QoL. The review findings are consistent with previous non-nurse-led intervention studies, which found that school-based asthma programs improved health outcomes and prevented complications (Horner et al., 2016; Rasberry et al., 2014; Suwannakeeree et al., 2016). However, not all of the interventions contributed to significant results. Indeed, there was a mix of statistically significant and nonsignificant findings. For example, only one study reported a significant reduction in asthma symptoms following the intervention (Isik et al., 2020), and one study found a significant difference in QoL postintervention (Harrington et al., 2018).

The review highlighted a positive impact on medication adherence and, therefore, symptom frequency among children. This positive impact was not significantly prominent among adolescents in Salisbury et al.’s (2002) study. This could be due to adolescents’ reluctance to go to the GP as recommended by the school nurse who advised participants who needed drug changes or delivery devices to contact their GP. The results in this study corroborate Persaud et al.’s (1996) findings of younger children being more vulnerable to visit the ER due to their asthmatic symptoms and are more likely to benefit from school nurse-led asthma programs; thus, indicating the significance of preventive health earlier in life. Ismaila et al. (2013) who systematically reviewed asthma burden in Canada emphasized this trend. They reported that rates of health care utilization outcomes (ER visits, hospitalization, and physician visits) are higher among children with asthma <18 years old.

The frequency and accuracy of medication administration through medication delivery devices to either prevent symptoms or for quick episode relief are important indicators of asthma management. The review reported a weakening of inhaler technique skills as early as 1 month after receiving education on best practice inhaler technique skills. This illustrates how improper inhaler technique is a major problem among asthmatic children (Román-Rodríguez et al., 2019). Suwannakeeree et al. (2016) made this point and noticed that the rate of ICS use among children with asthma at 6 months after the intervention was fewer than at 3 months after the intervention. While GINA guidelines recommended that inhaler technique should be assessed at every visit, this is challenging. One way of improving the inhaler technique is through systematic-standardized teaching by health care providers (Simoneau et al., 2020). As seen in this review, the studies varied regarding the number of follow-ups conducted, that is, some studies collected data in two separate occasions (one follow-up), while other collected data in three separate occasions (two follow-ups). School nurses are in the best position to conduct periodic skill assessment to prevent skill deterioration and promote the sustainability of learning.

As asthma is strongly linked to school absenteeism, studies supported school nurses’ role in reducing school absence among students with chronic conditions (Johnson et al., 2019; Moricca et al., 2013; Rodriguez et al., 2013). The American Academy of Pediatrics Council recommended establishing a relationship with school nurses to improve chronic condition management (Council on School Health, 2016). This is because school nurses play a proactive role in preventing school absence among younger children with asthma. In line with this, our review stressed the essential role the school nurse plays in preventing school absence among students with asthma. Despite reporting nonsignificant findings due to the small sample size, short assessment period, and variation in absence recording, the five included studies that investigated school absence reported a trend in the declining number of missed school days after the school nurse-led intervention, regardless of the intervention type. Furthermore, an important point to consider when assessing the asthma burden on school absence is the consideration of students’ and parents’ reports, as well as school health records (Johnson et al., 2019).

Addressing asthma knowledge among children and their parents is an important determining factor of asthma management. Our results demonstrated that educating parents and children was a major component of school nurse-led asthma interventions. The National Heart, Lung, and Blood Institute highlights the importance of involving children and their parents in asthma management (National Institutes of Health, 2012; van Bragt et al., 2015). However, in this review, only two studies examined asthma knowledge outcomes, one among children only and one among children and their parents. Despite being nonsignificant, the parent knowledge score showed an improvement (Persaud et al., 1996). Several school-based non-nurse-led asthma education programs addressed asthma knowledge among children and their parents (Grover et al., 2016; Horner et al., 2016; Kintner et al., 2015; Payrovee et al., 2014; Suwannakeeree et al., 2016). These studies showed improved knowledge scores postintervention. Agusala et al. (2018) recommended involving parents in asthma programs and indicated that parents reported increased knowledge in managing asthma symptoms and triggers. It is, therefore, clear that parents’ education was also associated with a reduction in health care utilization (Agusala et al., 2018). It is important to note that asthma knowledge can be improved through educational programs; however, knowledge improvement does not necessarily reflect an improvement in care practice.

It could be argued from our review that the consistency and sustainability of the intervention is a challenge. Trivedi et al. (2018) reported that relying on the school infrastructure and the existing school nurse service promoted the sustainability and reproducibility of the intervention. However, it should be noted that school nursing services are not available across the globe. This requires asthma intervention programs to be developed for school personnel such as schoolteachers. A systematic review by Coelho et al. (2016) emphasized such a trend by highlighting that asthma educational activities should include the whole school community. Hence, students, teachers, and school staff can recognize disease symptoms and management strategies (Coelho et al., 2016). While schoolteachers play an essential role in implementing school policies, the literature showed that their asthma knowledge was suboptimal (Lucas et al., 2012; Rodríguez Fernández-Oliva et al., 2010). To address such issues, some asthma education programs were developed for teachers in England, Italy, and the United States (Brooten et al., 2008; Goei et al., 2010; McWhirter et al., 2008). Also, a review by Al Aloola et al. (2014) discussed this point and reported that relying on nonhealth care personnel, such schoolteachers, to deliver asthma interventions may seem more sustainable; however, teachers’ limited time and training on asthma education could represent a challenge (Al Aloola et al., 2014). Similarly, Suwannakeeree et al. (2016) included five teachers in an asthma training program and supported that enhanced asthma knowledge among teachers provided them with an awareness of the child’s condition and enabled them to support children to manage their asthma symptoms.

Our review highlighted supervised asthma medication administration in school settings as a form of a school nurse-led asthma intervention. Salazar et al. (2018) emphasized that school-supervised asthma therapy improves asthma outcomes. Although successfully implemented, they suggested that medication administration by school nurses causes an uncompensated workload for school nurses (Salazar et al., 2018). Schools with limited nursing services can establish a system to maintain the administration of preventive asthma medications as seen in Halterman et al.’s (2018) study, where an asthma care coordinator who received additional asthma training was appointed to assist the school nurse in communicating with the physician, families, and school. This role promotes a therapeutic relationship between the school nurse and schoolchildren; thus, facilitating ongoing monitoring and education. An additional strategy that may help to reduce school nurse workload while implementing such programs is the electronic linkage between the hospital health record and school electronic health record, so that the school nurse is aware of any change in the treatment plan.

### School-Based Intervention Versus Nurse-Led Intervention

Many studies described school-based interventions targeting children, and these interventions showed improvement in asthma outcomes (Cicutto et al., 2013; Grover et al., 2016; Payrovee et al., 2014; Perry et al., 2018; Szefler et al., 2018). However, these programs are sometimes short-term, delivered by research teams, or require additional resources such as manpower or materials that could affect the sustainability of the intervention. Asthma intervention programs should be developed in schools to sensitize the school and community to self-management practice in the long run (Coelho et al., 2016). To enable practice sustainability and maintain asthma self-management skills, school nurses need to be involved in planning, implementing, evaluating, and leading the intervention (L. Carpenter et al., 2013; Isik et al., 2020). School nurses are leaders in the development of programs, policies, and procedures provided to students in the school system (NASN, 2016). Therefore, at the policy development and implementation level, school nurses act as change agents to promote the concept of self-management of chronic conditions such as asthma. Accordingly, school nurse-led programs are of particular importance to achieve sustainable evidence-based practice nursing care by creating a linkage between schools, hospitals, and home (L. Carpenter et al., 2013). As studies in this review did not elaborate on school nurses’ extent of involvement in intervention design, further research is needed to compare child health outcomes when asthma interventions are developed by school nurses versus implemented or led by school nurses.

### School Nurses’ Training Needs

The NASN framework for 21st Century School Nursing Practice includes leadership as one of the domains. Through school nurse-led interventions, school nurses contribute to the health and well-being of the students (Hoekstra et al., 2016) as well as to their academic development (Yoder, 2019). For school nurses to lead asthma interventions, there is a demand to assess their training needs to strengthen their competency. Ongoing education for school nurses based on a theoretical framework is recommended. Yet there is a dearth of qualitative studies that identify the training needs of school nurses in competency-based education programs (Shin & Roh, 2020).

In summary, different school nurse-led asthma interventions carried out in schools can improve child health outcomes. Training of school nurses specifically support positive practice sustainability at a reduced cost (L. Carpenter et al., 2013)

## Conclusion

Overall, this scoping review found that school nurse-led asthma intervention programs had a positive impact on children’s asthma-related outcomes. Several limitations identified in this review should be considered when planning a school nurse-led asthma program in the future. First, the included studies did not explore the effect a school nurse-led asthma program had on several other outcomes such as the number of exacerbations and asthma severity. Second, the dearth of evaluation strategies of school nurse-led programs requires investigation. It is not possible to draw a firm conclusion on the impact of a school nurse-led asthma program without an in-depth evaluation of such outcomes and a longer follow-up period of each outcome. Third, the wide variations in sample size, measurement tools, and the nature of school nurse-led asthma programs in the studies reviewed require a more rigorous type of research designed to better illustrate how nurse-led asthma programs are beneficial in reducing asthma-related outcomes. Most interventions included in our review were short-term, individual programs that lasted 1 year or consisted of four or less sessions. This reduced the impact of school nurse-led interventions or limited the opportunity to reinforce the instructions. Finally, the sustainability of interventions is influenced by the degree to which school nursing services are available, as the partnership between the clinical system (clinical setting) and the public system (school setting) promotes long-term asthma outcomes sustainability. Thus, the school nurse, as a member of an interprofessional team, can act as a linkage between the health sector and the educational sector. This provides an important insight that should be further explored in future research. Taken together, the findings highlighted in this review provide important practical implications in terms of ensuring the effectiveness of school nurse-led intervention programs addressing asthma among school-aged children. Thus, these insights can guide the future development and implementation of such programs, led by nurses, which will ensure positive asthma-related outcomes.
